# Research trend of circulating tumor DNA associated with breast cancer from 2012 to 2021: A bibliometric analysis

**DOI:** 10.3389/fonc.2022.1090503

**Published:** 2023-01-13

**Authors:** Zeqi Ji, Lingzhi Chen, Qiuping Yang, Huiting Tian, Jinyao Wu, Daitian Zheng, Jiehui Cai, Yexi Chen, Zhiyang Li

**Affiliations:** Department of Thyroid, Breast and Hernia Surgery, General Surgery, The Second Affiliated Hospital of Shantou University Medical College, Shantou, China

**Keywords:** circulating tumor DNA, breast cancer, bibliometric analysis, VOSviewer, Bibliometrics

## Abstract

**Background:**

Recently, ctDNA has become the focus for scientists with respect to personalized treatment, early screening, precise diagnosis, and prognosis of BC. This paper aims to use bibliometric analysis to investigate the research status and future trends in this field.

**Methods:**

All the related literature in the field of ctDNA and breast cancer was gathered from the Web of Science Core Collection. Data analyses were performed with R package Bibliometrics, VOS viewer 1.6.18, and online analysis in WoS. IBM SPSS (version 26.0) was used for statistical analysis.

**Results:**

A total of 739 publications, including 472 articles and 267 reviews, were retrieved. The overall number of articles published showed an upward trend. The United States has the largest number of published articles (266 papers) and citations (20,225 times). The most productive journal was Clinical Cancer Research. Cristofanilli M was the most prolific author, while Carlos C was the most cited one. The most frequent keywords excluding the search subject were “liquid biopsy”, “plasma”, “mutations”, “metastatic breast cancer”, “acquired resistance”.

**Conclusion:**

This article explored the application value of ctDNA in breast cancer with bibliometric analysis, offering an overall and intuitive understanding of this topic and revealing the study trends in the past ten years.

## Introduction

Breast cancer (BC) has been proven to be the most common malignancy with the highest mortality rate in women worldwide (1). Besides, BC is heterogeneous on the molecular level which was categorized into four subtypes according to different molecular features (2). And its therapeutic strategies vary according to the molecular subtypes. It is necessary to continuously assess the disease status and alternations of tumor biology (2). In this context, relying on serial biopsies is impractical, leading to a call for liquid biopsies which had the advantage on reflecting tumor heterogeneity (3). On this account, many scholars have been devoted to finding appropriate biomarkers for the diagnosis, treatment efficacy, prognosis, recurrence, and metastasis of BC.

Circulating tumor DNA (ctDNA) is a cancer-derived blood biomarker and also the widely studied circulating biomarker applied to liquid biopsy till now (4). With the development of the cancer genome project (CGP) and the wide application of next-generation sequencing (NGS), which effectively increase the sensitivity and specificity of ctDNA screening (5, 6), more and more researchers suggest ctDNA analysis as a promising biomarker for diagnosis, prognosis, and treatment of some malignancies (7, 8), such as lung cancer, colorectal cancer (CRC), BC, hepatocellular carcinoma (HCC) (9–11). Because of its capability to assess tumoral heterogeneity, ctDNA has become the focus for scientists with respect to personalized treatment, early screening, precise diagnosis, and prognosis of BC (12). O’Leary B, et al. have reported that the dynamic of ctDNA can be used to predict the long-term outcome of CDK4/6 inhibitors therapy in BC (13). However, there were still bottlenecks in the clinical application, for example, the blood collection, storage, and detecting techniques (14). What’s more, as time goes by, the somatic mutation related to cancer in the plasma may not be specific in people with cancer (8).

Bibliometrics is defined as the quantitative assessment of scientific outputs within a particular field using statistical methods (15, 16). Benefiting from the bibliometric analysis, scholars can collect information from retrieved academic literature, discuss their distribution characteristics and collaboration among authors and affiliations, evaluate the scientific quality and have a better understanding of emerging research topics and developments (15–17).

Although related articles in this field were widely discussed by researchers, there has been no bibliometric analysis found to comprehensively investigate the current progress, hotspots, and challenges with respect to ctDNA and BC. Based on this situation, this paper aims to use scientometric analysis to figure out the publications, authors, countries, journals, and cited references in the field of ctDNA and BC in the last decade, summarizing the hotpots and novel trends in this domain and providing references for future research direction.

## Materials and methods

### Data collection

**Figure 1:**
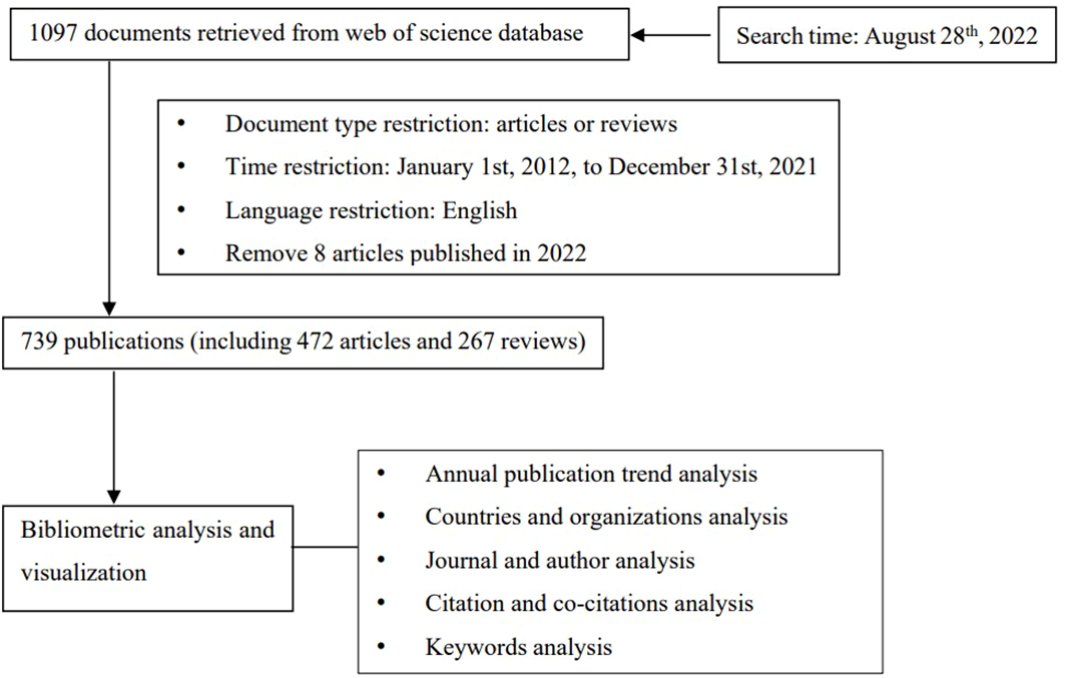
Flowchart of data collection and analysis.

Subject words were searched using the Medical Subject Headings (Mesh) database of Pubmed (https://pubmed.ncbi.nlm.nih.gov/) to make the retrieval more comprehensive and accurate. All the related literature about the relationship between ctDNA and breast cancer was gathered from Web of Science (WoS) Core Collection database with no limitation for citation index, including Science Citation Index Expanded (SCI-EXPANDED), Social Sciences Citation Index (SSCI), Arts & Humanities Citation Index (ESCI), Current Chemical Reactions (CCR-EXPANDED), Index Chemicus (IC). The query parameter was designed as followed: #1, TS=(“breast neoplasm*”) OR TS=(“breast tumor*”) OR TS=(“breast cancer*”) OR TS=(“mammary cancer*”) OR TS=(“mammary neoplasm*”) OR TS=(“breast carcinoma*”) OR TS=(“mammary carcinoma*”); #2, TS=(“Circulating Tumor DNA”) OR TS=(“Cell-Free Tumor DNA”) OR TS=(“ctDNA”); #3, “#1”, and “#2”. A total of 1097 pieces of literature were got in the preliminary search. And then, we did some screening: the publication year was refined from January 1st, 2012 to December 31st, 2021, the document type was set to articles or reviews, and the language was restricted to English. And we also excluded the articles published in 2022. All the searches were performed on August 28, 2022 and finally, we got 739 publications, including 472 articles and 267 reviews, accounting for 63.870% and 36.130%, respectively. The flow diagram of data collection and filtering was shown in **Figure 1**.

### Data analysis

With the function of “analyze results” in WoS, we did the preliminary analysis of the retrieved literature, including author, publication year, document type, research direction, country, affiliated institution, publisher. The function of “citation report” was used to acquire the information about the number and citation frequency of each paper.

Then, the information of the literature retrieved by WoS was exported in plain txt format and imported into the tools of biblioshiny (the shiny app for bibliometrix) and VOSviewer for further analysis of annual production, authors, countries, journals, institutions, keywords, and citations.

Bibliometrix package is a scientometric analysis software based on R language. In this study, we used the biblioshiny website with R language (version R 4.1.0) to analyze the bibliographic information of retrieved literature. An overview of basic information contained the time span, annual growth rate, average citations per doc, document contents, document types etc. Descriptive analysis and visual presentation about the annual production, average citation per year, contribution of countries, organizations, and journals, authors publications and impact were acquired. The author impact was evaluated by Hirsch h-index, g-index, m-index. The h-index indicated the impact of a researcher’s scientific outputs, defined as the largest number of articles published by a researcher which have been cited at least h times (18, 19).

VOSviewer (version 1.6.18), a computer program for constructing and visualizing bibliometric maps in an intuitive and intelligible view (20). In this study, VOSviewer was applied to perform the co-authorships analysis of countries, organizations, and authors, bibliographic coupling analysis of journals, co-citation analysis of cited reference, co-occurrence analysis of keywords. We also used this software to gain the total link strength among different countries, organizations, and authors.

IBM SPSS (version 26.0) was used for statistics analysis. We set the data type as numerical variables and used Spearman correlation analysis to analyze the correlations between selected variables. All the tests were two-sided, and p-values < 0.05 were considered statistically significant.

## Results

### Analysis of annual publication trends and average citations

**Figure 2:**
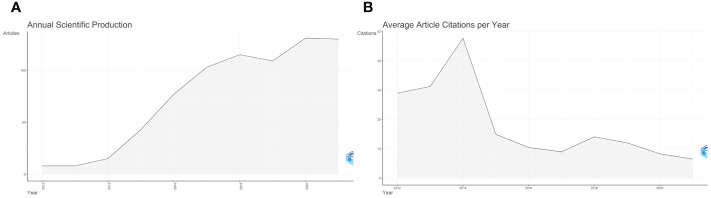
Annual publication and average article citations per year of ctDNA in the field of breast cancer from 2012 to 2021. **(A)** Annual publication from 2012 to 2021 in this field; **(B)** Average article citations per year from 2012 to 2021 in this field.

A total number of 739 documents contributing to this research field from 2012 to 2021 was obtained from WoS. **Figure 2A** showed the change in annual publication volume from 2012 to 2021 (r^2^ = 0.973, [CI, 0.806 to 1.000]; p < 0.001). From 8 articles published in 2012 to 130 articles published in 2021, the overall number of articles published showed an upward trend, with an average annual growth rate of 36.31%. The highest number of publications was produced in 2020 (131, 17.72%). From 2012 to 2014, there were few articles in this field, with a total of 31 articles published in the three years, reflecting that this field arouse little concern from the researchers at that time. From 2015 to 2018, the annual output increased rapidly, with a median annual output growth rate of 34.65% (2015 had the highest output growth rate of 65.11%), indicating a sharp rise in academic attention and study in this field during this period. There was a small decline in publishing in 2019 but it remained high. From 2019 to 2021, the number of publications continued to increase slowly, indicating that researchers or scholars have kept a watchful eye on this field.

Among all the retrieved articles, a total of 37,987 citations were received and the average citation frequency of each article was 51.4 times. **Figure 2B** showed the average citations per year from 2012 to 2021. The number of citations increased sharply in 2014, reaching the highest peak (n=47.68) in the past decade. In 2018. the citations per article reached another small peak (n=14.1) and then it continued to decline until it reached the lowest average citation frequency in 2021 (n=6.47).

### Analysis of country and organizations

Country analysis helps to reveal the geographical distribution of relevant papers in the field. There are 52 countries involved in the 739 retrieved documents contributing to this research area. The top 10 cited countries according to the rank of total citations were performed in **Table 1**. We can learn that United States (US) has the largest number of published articles (266 papers), accounting for 35.99%; and then China (148 papers, 20.02%) followed by Italy (86 papers, 11.63%) and the United Kingdom (UK) (73 papers, 9.87%). The number of citations of a country’s published literature is also an important factor to measure its influence on the field. US is still the top cited country with 20,225 times, followed by UK (8,377 citations), Italy (7,913 citations). Australia (7,338 citations), Germany (6,856 citations). However, in terms of total citations per production, Sweden (436 citations), Australia (236.7 citations), and Brazil (192.1 citations) took the top 3 countries, respectively. Obviously, the influence of US on this field is beyond doubt. Moreover, it is worth noting that although the publications of Italy, UK and Australia is less than 100, their publications are cited more frequently than those of China.

**Table 1:** Top 10 cited countries contributing to this research area.

Country	Production	%/of papers	Total citations	TC/P
USA	266	35.994	20225	76.033
UK	73	9.878	8377	114.753
Italy	86	11.637	7913	92.012
Australia	31	4.194	7338	236.710
Germany	59	7.984	6856	116.203
France	57	7.713	4448	78.035
China	148	20.027	3387	22.885
South Korea	20	2.706	3338	166.9
Sweden	7	0.947	3052	436
Brazil	15	2.030	2882	192.133

TC, the total number of citations a country has received.

TC/P, the average number of citations per production in a country.

**Figure 3:**
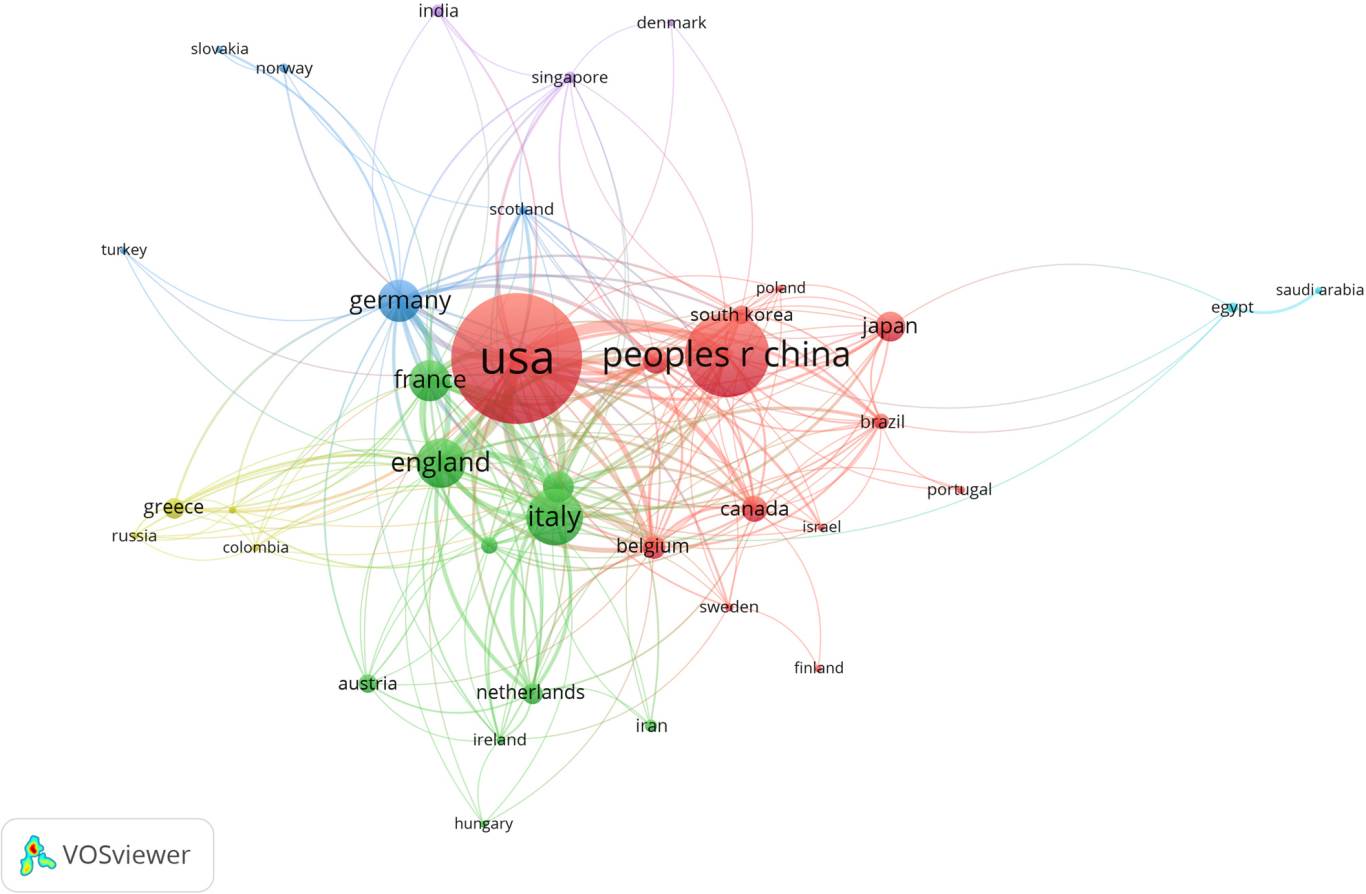
Co-authorships analysis of countries in network visualization map.

And then VOSviewer was used to conduct co-authorships analysis of countries with the threshold set as 3 documents. The size of nodes represents the quantity of literature published in each country, and the larger the node is, the more the country produced. The connection between nodes represents the cooperation between countries, and the thicker the line, the closer the cooperation between countries. It is apparent that the United States was the central country in this field, and has a close cooperation with China, Italy, UK, France, and Germany (**Figure 3**). The total intensity of links of USA (212) is significantly higher than other countries. In addition, although China had a higher academic output, the total link intensity (69) was less than Italy (129) and UK (124).

**Figure 4:**
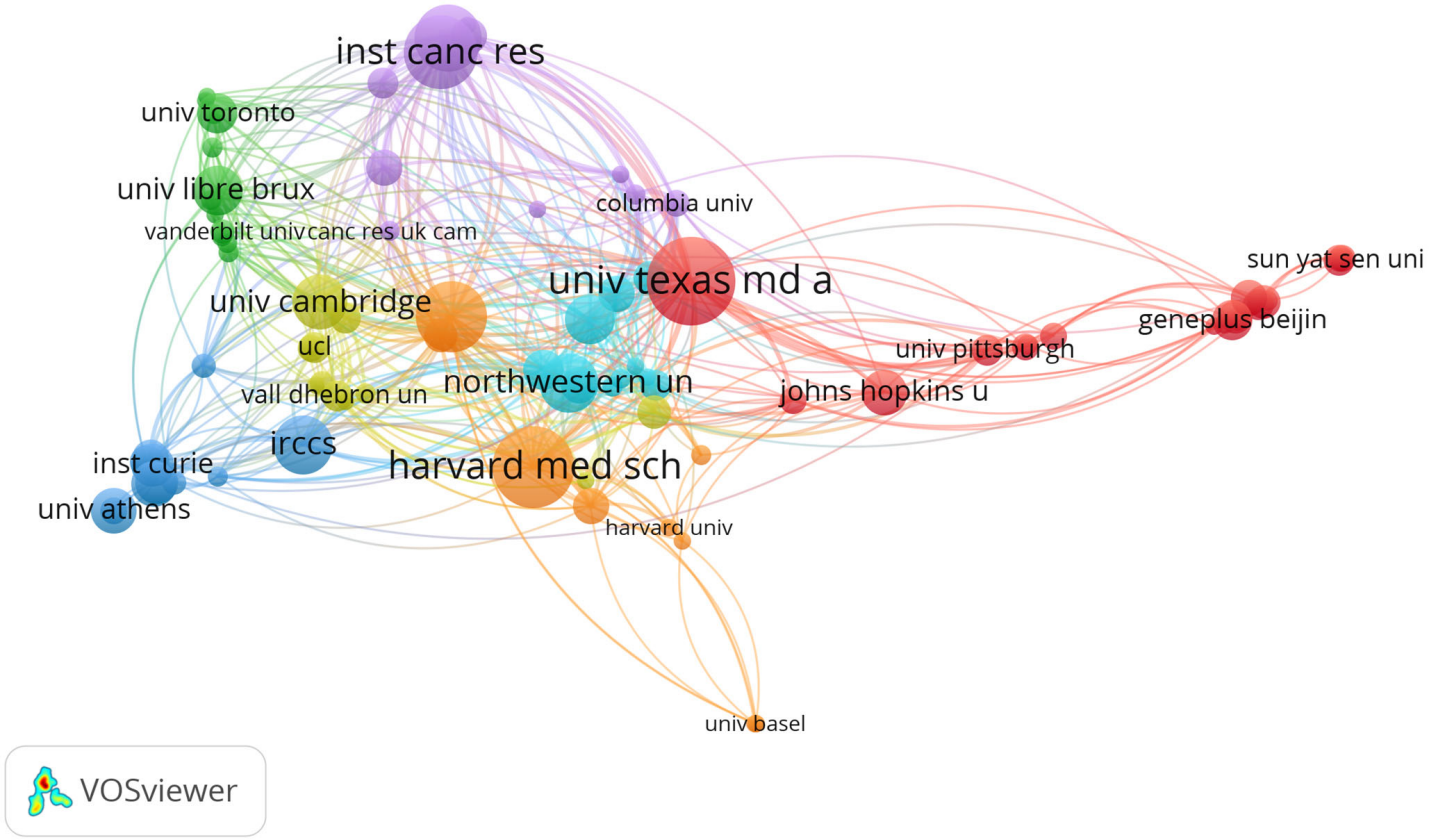
Co-authorships analysis of organizations in network visualization map.

Setting a threshold of 5 documents of an organization in the co-authorships analysis of organizations, and we got 91 of 1375. **Figure 4** provides a network visualization map of the major organizations; Among them, the top 5 for publications were University of Texas MD Anderson Cancer Center (n=28), Harvard Medical School (n=26), Institute of Cancer Research (n=23), Memorial Sloan-Kettering Cancer Center (n=22) and Royal Marsden Hospital (n=21). However, the top 3 with most citations were Memorial Sloan-Kettering Cancer Center (6219 citations), University of Turin (4259 citations), and University of Cambridge (4085 citations). The top three in total link strength were Institute of Cancer Research (43), Royal Marsden Hospital (43), and Memorial Sloan-Kettering Cancer Center (39) (**Table 2**). Notably, of the top 10 most productive organizations, five are from the United States, three are from the United Kingdom, and the other two are from Italy and Belgium.

**Table 2:** Top 10 organizations based on publications.

Organization	Publication	Total citations	Total link strength	Country
University of Texas MD Anderson Cancer Center	28	3093	68	USA
Harvard Medical School	26	1382	55	USA
Institute of Cancer Research	23	2695	68	UK
Memorial Sloan Kettering Cancer Center	22	6219	67	USA
Royal Marsden Hospital	21	2608	65	UK
IRCCS	18	636	29	Italy
Northwestern University	18	539	37	USA
Cambridge University	17	4085	42	UK
Dana-Farber Cancer Institute	15	1710	44	USA
Vrije Universiteit Brussel	15	957	30	Belgium

### Analysis of journal and research categories

**Figure 5:**
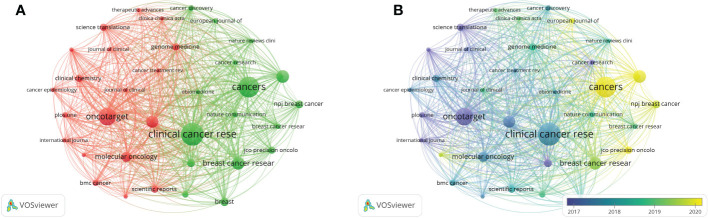
Bibliographic coupling analysis of sources in this fields. **(A)** Bibliographic coupling analysis of journals in network visualization map. **(B)** Bibliographic coupling analysis of journals in overlay visualization map.

The analysis of journals in related research fields can provide references for researchers to get better submission selection. There are 300 journals active in the field; By setting 5 as the minimum number of documents of a source, 35 of them meet the thresholds and the visualization of sources analysis was got (**Figure 5A**). Clinical Cancer Research got the largest number of publications (n=32), followed by Cancers (n=29), the third to fifth were Oncotarget (n=24), Breast Cancer Research and Treatment (n=17), and Frontiers in Oncology (n=17). The total number of articles published in the top 10 journals was 179, accounting for 24.22%. Science Translational Medicine had the most citations (5205 citations) with only 9 documents. And the next were Clinical Cancer Research (2489 citations), Journal of Clinical Oncology (2138 citations), Cancer Discovery (1761 citations) and Annals of Oncology (1297 citations). The h-index is an indicator reflecting the quality of scientific publications (21). In this study, Clinical Cancer Research ranked first with the h-index of 24, and followed by Oncotarget and Annals of Oncology, with the same h-index of 16, and Molecular Oncology (h-index = 11). Of the top 10 journals based on publications, Clinical Cancer Research was the first to start publishing articles in this field in 2012 and 70% of them started publishing in 2014-2015. (**Supplementary Table S1**).

From the overlay visualization map of sources analysis in VOSviewer (**Figure 5B**), as the blue color represent the earlier average publication year while the yellow color represents the more recent average publication year, we found that Science Translational Medicine, Cancer Research, and Oncotarget have been active in this field at least 5 years ago. Moreover, although Cancers, and Frontiers in Oncology have become active in the past 2 years, the number of published articles has already ranked among the top 5.

There are 46 research categories and 70 publishers involved in the retrieved literature. The top 5 subject categories and publishers based on the number of publications are listed in **Supplementary Table S2**. The most represented one is “Oncology” (n=429, 58.051%), accounting for more than half of all documents. Followed by “Research Experimental Medicine” (n=65, 8.796%) and “Cell Biology” (n=60, 8.119%). Regarding publishers, “Springer Nature” ranked the first (n=155, 20.974%), followed by “Elsevier” (n=113, 15.291%), “Amer Assoc Cancer Research” (n=57, 7.713%), and “Mdpi” (n=51, 6.901%).

### Analysis of authors

Through statistical analysis, 5017 authors contributed to all the 739 documents, 15 among them were single-authored documents. The average number of authors per document was 6.78, and the average number of co-authors per document was 9.56. Cristofanilli M from US was the most prolific author (19 articles), followed by Turner NC, from UK (n=17) and Ma F, from US (n=16) (**Table 3**). Ranked by h-index, Turner NC was the top 1 (h-index = 15), and then Garcia-murillas I (h-index = 13), Hhrebien S (h-index = 12), Cristofanilli M (h-index = 11). M-index is an indicator for “scientific quality” which was corrected for the time of scientific activity (21). Turner NC and Garcia-murillas I also occupied the top two places, with the m-index of 1.875 and 1.675, respectively. Cristofanilli M ranked No.3 (m-index = 1.571) and followed by Hrebien S (m-index = 1.500). In terms of the most cited authors in this fields, the results were a little different. Carlos C ranked first, with 7 published papers receiving 3723 citations, indicating that his literature has made a significant contribution to the development of the field. And it was followed by Nitzan R (3520 citations), and Sarah-Jane D (2405 citations), with publication count less than 10 articles, suggesting a great impact of each of their literature on this field.

**Table 3:** The top 10 with most published authors in this field.

Highly published Authors	NP	TC	h-index	g-index	m-index
CRISTOFANILLI M	18	999	11	18	1.571
TURNER NC	17	2491	15	17	1.875
MA F	16	306	9	16	1.286
BIDARD FC	15	870	10	15	1
GUAN YF	15	282	9	15	1.286
PIERGA JY	15	541	9	15	1
GARCIA-MURILLAS I	14	1715	13	14	1.625
XU BH	14	284	8	14	1.143
YI ZB	14	298	9	14	1.286
YI X	13	333	9	13	1.286

NP: number of publications.

TC: total cications.

**Figure 6:**
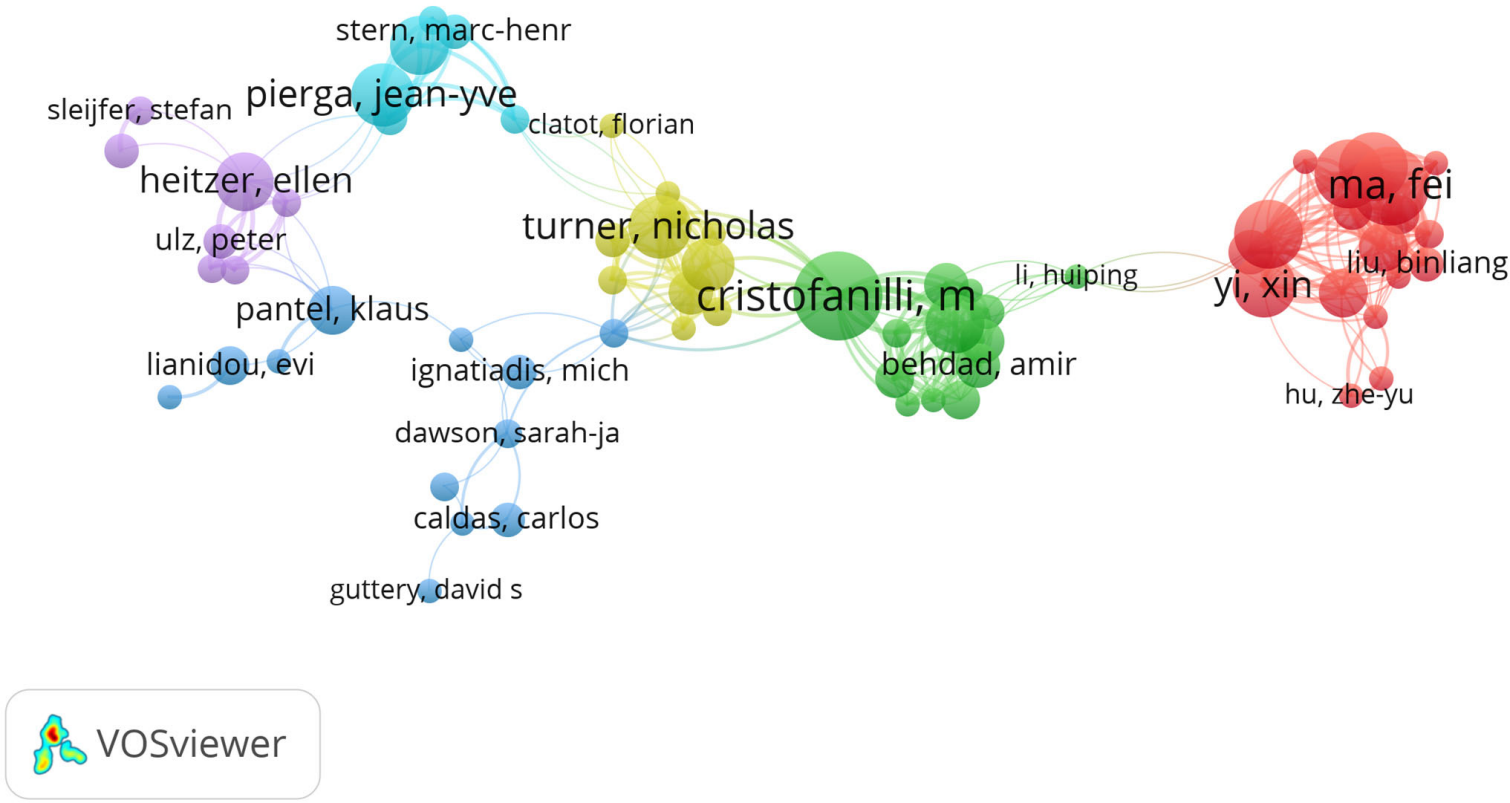
Co-authorship analysis between authors in network visualization.

Co-authorship analysis between authors was conducted using VOSviewer, and 79 co-authors wrote more than 5 articles. Preserving the largest set of 67 connected items and we got the Visualization Map (**Figure 6**). Cristofanilli M had the most total link strength (100), and was followed by Ma F (99), Yi ZB (96), Xu BH (90), Guan XW (81). The co-citation analysis of cited authors is shown in **Supplementary Figure S1**, where a total of 15 authors were cited more than 150 times. The top five cited authors are Dawson SJ (321), Diehl F (302), Bettegowda, C (275), Garcia-Murillas I (271), and Mmurtaza M (243).

### Analysis of citations

We used bibliometrix to analyze the specific information of the top 10 documents in this field (**Supplementary Table S3**). All these articles were all published between 2012 and 2018. 70% of them published before 2015. Among them, the most cited paper was “Detection of circulating tumor DNA in early- and late-stage human malignancies (Bettegowda, C 2014)” published in Science Translation Medicine Right, with the total citations 2655, analyzing the early detection of DNA in human malignancies (22). Dawson et al. published an article in The New England Journal of Medicine in 2013 titled “Analysis of Contaminated Tumor DNA to Monitor Breast Cancer”, with the second highest citations (1,488). In this article, it revealed that contaminated tumor DNA is a highly sensitive and informative biomarker of breast cancer (23). The third is “Liquid Biopsies: Genotyping Circulating Tumor DNA” published by Diaz, LA and Bardelli, A in the Journal of Clinical Oncology in 2014, receiving 1374 direct citations. This paper describes the sensitivity and accuracy of ctDNA based liquid biopsy to type the mutated genes of tumor cells, which is of great significance for clinical research (24). From the perspective of content, these three papers are related to the theoretical basis of ctDNA as a monitoring index for the detection of early breast cancer, which are representative and influential basic papers in this field and have far-reaching influence and significance for subsequent research.

**Figure 7:**
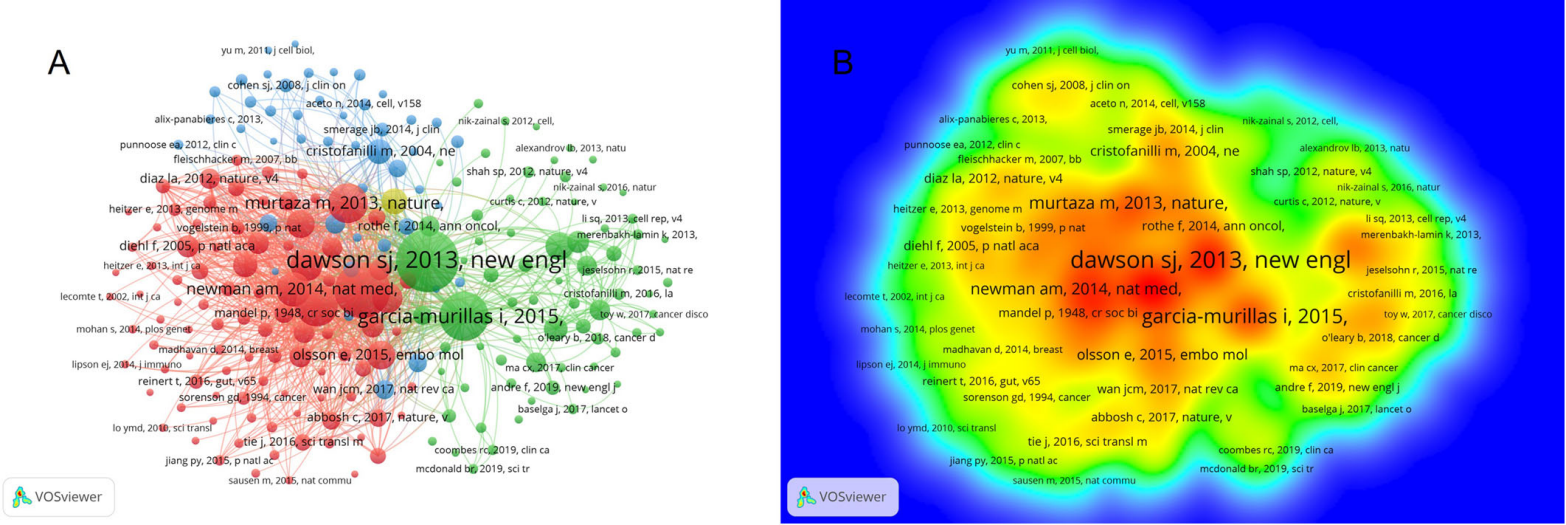
Co-citations analysis of cited references in this research fields. **(A)** The network visualization of co-citations analysis of cited references. **(B)** The density visualization of co-citations analysis of cited references.

From the co-citations analysis of cited references in VOSviewer, we can learn that 215 of the 24894 co-cited references had been cited at a minimum number of 20 times. In the network visualization, all the cited references are divided into four clusters and larger node represents more reference cited (**Figure 7A**). The top cluster with 96 items is shown in red, indicating the most interesting research area. The redder color means more citations in the density view (**Figure 7B**). **Supplementary Table S4** listed the top ten most co-cited references. The top five cited references are Dawson SJ (2013; 310 citations), Bettegowda C (2014; 266 citations), Garcia-murillas I (2015; 228 citations), Diehl F (2008; 189 citations), Murtaza M (2013; 173 citations).

### Analysis of keywords

291 keywords were obtained to perform keyword analysis by setting the occurrence frequency to more than 5. With the function of clustering on VOSviewer, all keywords were divided into four clusters shown in different colors (**Supplementary Figure S2A**). The size of an item’s circle and label is proportional to its importance. The items in the same cluster with the same color mean higher correlation with each other (20). The red cluster and the green cluster are the two main clusters in this field. The red cluster contains 117 keywords, mainly including circulating tumor DNA, breast cancer, mutations, therapy, heterogeneity and so on. The green cluster contains 108 keywords, including cell-free DNA, plasma, acquired resistance, and so on. The blue cluster is the third largest cluster with 65 items, including liquid biopsy, survival, metastatic breast cancer (MBC), circulating tumor cell and so on. In the density visualization map, the color of an item depends on its frequency of occurrence, which is easy for us to pay attention to the most important areas in a map (20). The redder color means the heavier weight. As shown in **Supplementary Figure S2B**, the most frequent keywords excluding the search subjects were “liquid biopsy” (n=196), “plasma” (n=112), “mutations” (n=101), “metastatic breast cancer” (n=100), “acquired-resistance” (n=98) and so on. What’s more, the overlay visualization map showed the average publication year in which keywords appears in these publications (**Supplementary Figure S2C**). Yellow means the later it occurred in the articles. We can learn that the focus of research areas ranged from the expression of ctDNA genes in various cancers to the value of peripheral blood ctDNA as a biomarker and then to the diagnostic and prognostic value of ctDNA in BC.

The word or phrases that frequently appear in the title of an article’s cited reference were extracted and defined as keywords plus, which is used to identify research topics more descriptively and objectively (25). From the co-occurrence analysis of keywords plus analysis in VOSviewer (**Supplementary Figure S3A**), except from the search subjects, the top 5 with most cited keywords were “cell-free DNA” (n=140), “plasma” (n=105), “metastatic breast cancer” (n=100), “acquired-resistance” (n=98), and “mutations” (n=98). The overlay visualization map of keywords plus analysis showed the most recent keywords, including “liquid biopsy”, “cell-free DNA”, “ESR1 mutations”, “survival”, “resistance”, “double-blind”, “palbociclib”, and “fulvestrant”. (**Supplementary Figure S3B**).

## Discussion

It remains a global challenge for cancers to the early diagnosis and effective recurrence detection and therapeutic evaluation in spite of the progress made in the treatment (26). In this case, a calling for a specific and effective biomarker has arisen, thus achieving the detection of breast cancer at an early and treatable stage (27).

The research scope of this paper is set from 2012 to 2021. In 2012, only 8 articles were published in the research field of ctDNA and BC. Since 2015, the number of articles published in this field has grown rapidly, reaching 103 articles in 2017, exceeding 100 articles every year since then, and reaching a peak of 131 articles in 2021. It showed a burst of interest and sustained attention on this field. As of the retrieval date, the papers analyzed in this study have been cited for 37,987 times, with an average of 51.4 times for each paper. Since 2012, the number of citations has increased 258 times, and the literature published in 2014 has the highest number of citations per paper, reflecting the increasing attention and importance of this field. What’s more, it is indicated that the articles published in 2014 have laid an important foundation for the development of this field. Among the top 10 most cited documents, 90% verified that ctDNA has important clinical significance for the identification of early cancer (22–24, 28–33), and mainly discussed the significance of ctDNA for the diagnosis, prognosis prediction and management of systemic therapy of cancer. From the perspective of content, the top 3 most cited papers are related to the theoretical basis of ctDNA as a monitoring index for the detection of early breast cancer, which are representative and influential basic papers in this field and have far-reaching influence and significance for subsequent research. Two of these papers demonstrated that ctDNA could be used to identify and monitor MBC and identify breast cancers at high risk of recurrence (23, 30). Interestingly, the review of ctDNA published by Merker et al. concluded that there is insufficient evidence to demonstrate the clinical efficacy and utility of ctDNA in cancer screening, treatment monitoring or residual disease monitoring, and advanced cancer (34). This may be related to the fact that the latter authors put more emphasis on the feasibility of practical clinical application.

In total, 52 countries contributed to the field. The United States is undoubtedly the dominant country in this field, with the largest total number of publications, total number of citations and total link strength. Further analysis displayed that although the number of articles published in the UK and Italy was less than that in China, the number of citations and the total link strength of their articles were significantly higher than that of China, indicating that the academic influence of a country is not only reflected in the number of articles published, but also needs to be comprehensively evaluated by the number of citations, cooperation with other countries and link strength. China should encourage innovative inventions and research, not just the quantity of published literature.

On the top10 for publications of 300 journals, Clinical Cancer Research or Oncotarget was the first choice for many researchers to submit articles five years ago. In the past two years, Cancers and Frontiers in oncology have become the focus of submission for scholars, with a sharply rising number of publications. Meanwhile, in the past three years, breast cancer related journals such as Breast Cancer Research and Treatment, NPJ Breast cancer and Breast Cancer Research have also achieved a satisfactory volume of scientific publications in this field. It can be speculated that the relationship between ctDNA and BC is becoming more and more popular among these special journals related to breast cancers.

Cristofanilli M (n=19), Turner NC (n=17), Ma F (n=16), Bidard FC (n=15), Guan YF (n=15) were top five authors by number of publications. Besides, Turner NC had the highest h-index. His first paper on this subject was published in 2015, which demonstrated that targeted sequencing of ctDNA can be used to detect minimal residual disease in BC and thus more accurately predict genetic events of metastatic recurrence (30). It will be helpful for guiding adjuvant therapeutic intervention of early breast patients at high risk of metastasis and recurrence. The author with the highest numbers of citations is Carlos C. His first paper on this subject, published in 2012, showed that tagged-amplicon deep sequencing (Tam-Seq) was a feasible method for noninvasive detection of both abundant and rare mutations of ctDNA, which lays a foundation for personalized genomics for “liquid biopsy” (31). In 2013, Carlos C further proved that ctDNA show sensitivity and specificity in the detection of MBC (23). Interestingly, we notice that it is divided into six items in the co-authorship analysis between authors, the links within items are strong while the links between items are sparse.

Keyword analysis can be used to present emerging topics and predict future research directions in this field. From the perspective of keyword analysis, the research field of ctDNA and BC is mainly divided into three parts. At the early stage, the development of ctDNA to detect and monitor tumor burden has been widely discussed and it is indicated that ctDNA is feasible to be used to detect tumor dynamics in some solid cancer patients (22), such as MBC (23), pancreatic cancer (35), CRC (36). Furthermore, sequencing technologies of ctDNA to monitor cancer mutations are constantly evolving. Tim F. et al. have developed a method for Tam-Seq and used it to identify cancer mutations in ctDNA, with sensitivity and specificity of > 97% (31). With more detailed studies have been carried out, researchers are concerned about the clinical implications of gene mutations in ctDNA for cancer evolution and treatment modification. David C. et al. have verified that a high frequency of estrogen receptor alpha gene (ESR1) mutations occurred in ctDNA from patients with MBC by NGS (37). On this basis, further study was conducted to explore the significance of detecting ESR1 mutations in guiding personalized treatment decisions for MBC (38). Some researchers have focused on tumor heterogeneity and suggested that ctDNA can be used to predict treatment outcome by assessing tumor heterogeneity (39). Recently, the main attention of the field has been shifted to evaluate the sensitivity and specificity of liquid biopsy in a large clinically relevant cohort. Studies have showed that ctDNA was identified as a promising biomarker for real-time efficacy evaluation of BC, recurrence risk stratification, and personalized follow-up (40). Nonetheless, subsequent clinical trials are still needed to verify the clinical feasibility of performing liquid ctDNA biopsies on a large scale. From the keywords plus analysis, we can infer the emerging themes like “liquid biopsy”, “cell-free DNA”, “ESR1 mutations”, “survival”, “resistance”, “double-blind”, “palbociclib”, and “fulvestrant”, is cited until 2019, which may become hot topics in the future and needs further research.

“cell-free DNA” (cfDNA) is thought to be secreted through apoptosis and necrosis, and the kirsten rat sarcoma viral oncogene (KRAS) mutations detected in cfDNA were found of tumor origin, giving rise to the term ‘ctDNA’ (29). In the past few years, “liquid biopsy” has been used to isolate cancer derivatives circulating in blood or other body fluids, longitudinally detect cancer progression and residual disease after treatment, and predict disease recurrence at an early stage (41). In addition, the development of high-sensitivity techniques provided important information for the diagnosis, disease progression and treatment response of BC (42). However, limitations remain since ctDNA represents a small fraction of total cfDNA and is not shed by all tumors, giving challenges in the reliability and reproducibility of detecting low-mutation allele mutations (41, 43). What is more, the acquisition and analysis of ctDNA samples was not standardized and some scholars proposed to develop multi-parameter assays and combine different analytes to improve the prognosis of malignant tumors (44). Whereas, there is still insufficient of clinical trials to prove the efficacy of liquid biopsy (41). Therefore, further clinical trials are needed to improve its accuracy and sensitivity, which may be an interesting topic for continued discussion in the future.

The keywords of “ESR1 mutations”, “survival” and “resistance” are closely related to each other. “ESR1 mutations” are common in patients with ER-positive MBC during aromatase inhibitors (AI) medications, especially after the development of hormone resistance (45). Early identification of ESR1 mutations in ctDNA can predict the disease evolution of patients with MBC, manage clinical treatments (46). Lauren D et al. have evaluated ctDNA dynamics as promising biomarker for medicine efficacy and prognostic prediction for progress-free survival in MBC (47). In addition, ctDNA analysis was also conducted to identify potential resistance mechanisms to CDK4/6 inhibitors in estrogen receptor positive BC (48). Further confirmation of the clinical effectiveness of ctDNA analysis are required. Given advanced cancer assessment provided by ctDNA testing, related research on neoadjuvant chemotherapy for early-stage high-risk breast cancer and survival analysis for advanced MBC has been carried out recently, which further verify facilitation of the prediction of pathological complete response (pCR) and the risk of metastatic recurrence, as well as real-time assessment of treatment response (49–51). Circulating DNA was detected for molecular analysis to evaluate the prognostic value in a multicenter, “double-blind”, phase 3 randomized controlled trial (PALOMA-3), which confirmed the significant improvement of “fulvestrant” plus “palbociclib” in overall survival and progress-free survival compare with fulvestrant plus placebo (52, 53). It may also trigger more clinical trials on survival analysis of different medication treatments in advanced, metastatic breast cancer in the future.

There were still limitations in the present study. Firstly, we only used the web of science database to search for related publications, which may exclude some influential papers included from other databases, such as PubMed, Embase, and Scopus, resulting in selection bias. However, WoS is still the most extensively used tool for bibliometric analysis, with rich information of distribution of authors, countries, journals, organizations, and citations. Secondly, the research criterion of language type was limited to English. It is possible to cause a lack of high-impact articles written in other language. Thirdly, the articles published this year have not been bring into study, the result of this research is only applicable to the time point until August 28, 2022. Despite these, we believe that our finding can provide valuable advice on future development for researchers in this field.

## Conclusions

In this study, we explored the application value of ctDNA in breast cancer with bibliometric analysis, offering an overall and intuitive understanding of this topic and revealing the study trends in the past ten years. Publications related to this field showed a rapidly upward trend. The keyword analysis indicated that the current focus of this field may be the detection of genetic mutation in ctDNA to predict disease progression and treatment effectiveness of BC. Further clinical trials of ctDNA and the standardization and clinical feasibility of liquid biopsy detection are future attention.

## Data availability statement

The original contributions presented in the study are included in the article/**Supplementary Material**. Further inquiries can be directed to the corresponding authors.

## Author contributions

JC, ZL, and YC participated in the conception and design of the study. QY and HT collected the data. JC and LC provided the guiding suggestions on application usage and data analysis. ZJ, JW, and DZ were involved in data analysis and drafted the manuscript. ZJ was the major contributor to writing the article. LC revised the final version of the manuscript. All authors contributed to the article and approved the submitted version.

## Glossary

**Table:**

BC	breast cancer
ctDNA	crculating tumor DNA
CGP	cancer genome project
NGS	next-generation sequencing
CRC	colorectal cancer
HCC	hepatocellular carcinoma
Mesh	Medical Subject Headings
WoS	Web of Science
SCI-EXPANDED	Science Citation Index Expanded
SSCI	Social Sciences Citation Index
ESCI	Arts & Humanities Citation Index
CCR-EXPANDED	Current Chemical Reactions
IC	Index Chemicus
h-index	Hirsch index
Tam-Seq	tagged-amplicon deep sequencing
MBC	metastatic breast cancer
ESR1	estrogen receptor alpha gene
cfDNA	cell-free DNA
KRAS	kirsten rat sarcoma viral oncogene
AI	aromatase inhibitors
pCR	pathological complete response
