# Supplementary material for: Research trend of circulating tumor DNA associated with breast cancer from 2012 to 2021: A bibliometric analysis
Source: Front Oncol. 2023 Jan 13;12:1090503. doi: 10.3389/fonc.2022.1090503 (PMC9880534; doi:10.3389/fonc.2022.1090503)
Supplement: Supplementary file 1 [file DataSheet_1.pdf]

# *Supplementary Material*

## 1 Supplementary Figures

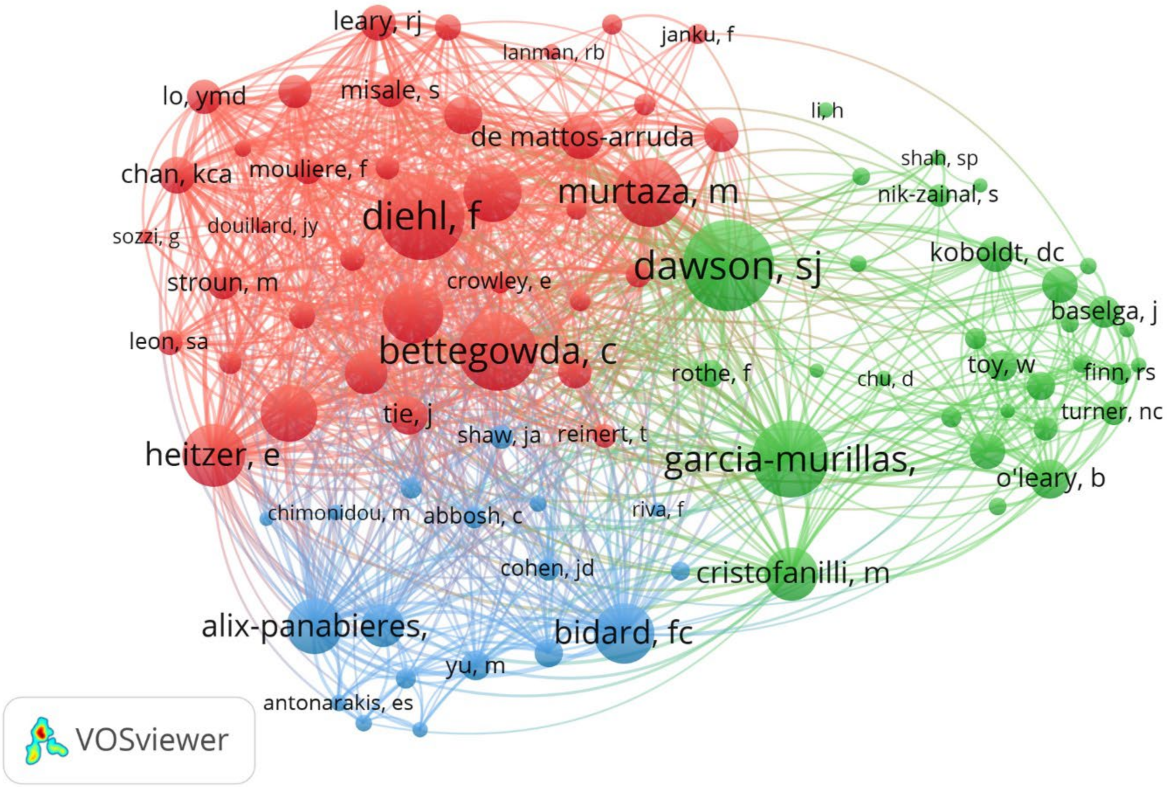


Supplementary Figure S1. Co-citation analysis of authors with minimum citations over 20 times.

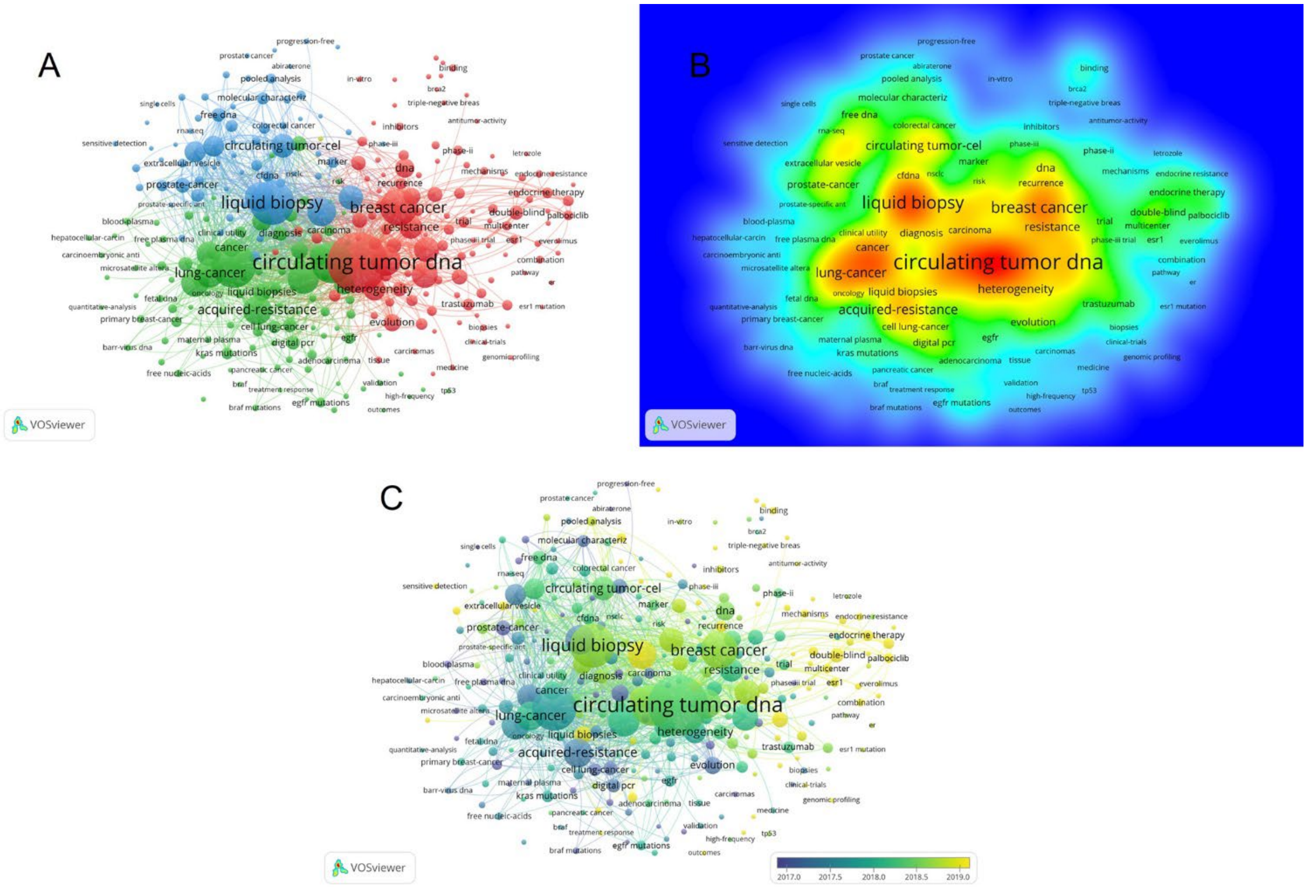


Supplementary Figure S2. Co-occurrence analysis of all keywords. (A-C) The network, density, and overlay map of co-occurrence of keywords analysis.

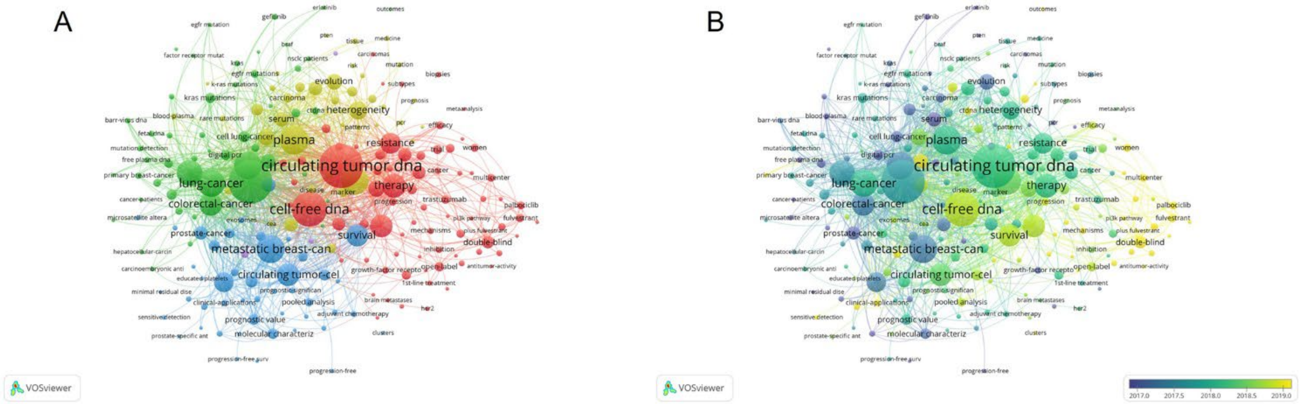


Supplementary Figure S3. Co-occurrence analysis of keywords plus. (A-B) The network and overlay map of co-occurrence of keywords plus analysis.

## 2 Supplementary Tables

Supplementary Table S1 | Top 10 journals based on publications

| Journals | Publication | Total citations | PY_start | h-index |
|---|---|---|---|---|
| Clinical Cancer Research | 32 | 2489 | 2012 | 24 |
| Cancers | 29 | 203 | 2014 | 8 |
| Oncotarget | 24 | 797 | 2015 | 16 |
| Breast Cancer Research and Treatment | 17 | 239 | 2015 | 7 |
| Frontiers in Oncology | 17 | 182 | 2017 | 6 |
| Annals of Oncology | 16 | 1297 | 2014 | 16 |
| Molecular Oncology | 13 | 650 | 2015 | 11 |
| Breast | 11 | 160 | 2015 | 5 |
| Expert Review of Molecular Diagnostics | 10 | 130 | 2015 | 8 |
| NPJ Breast Cancer | 10 | 136 | 2017 | 6 |

PY_start: the year that starting publication

Supplementary Table S2 | Top 5 active research subject categories and publishers

| Rank | Research subject categories | Documents | Publishers | Documents |
|---|---|---|---|---|
| **1** | Oncology | 429 | Springer Nature | 155 |
| **2** | Research Experimental Medicine | 65 | Elsevier | 113 |
| **3** | Cell Biology | 60 | Amer Assoc Cancer Research | 57 |
| **4** | Genetics Heredity | 42 | MDPI | 51 |
| **5** | Biochemistry Molecular Biology | 39 | Whiey | 42 |

Supplementary Table S3 | The top 10 documents with the most citations in this field

| Documents (Author/year/journal/Volume/Page/DOI) | TC |
|---|---|
| Bettegowda C, 2014, SCI TRANSL MED, V6, DOI 10.1126/scitranslmed.3007094 | 2655 |
| Dawson SJ, 2013, NEW ENGL J MED, V368, P1199, DOI 10.1056/NEJMoa1213261 | 1488 |
| Diaz LA, 2014, J CLIN ONCOL, V32, P579, DOI 10.1200/jco.2012.45.2011 | 1374 |
| Cohen JD, 2018, SCIENCE, V358, P926, DOI 10.1126/science.aar3247 | 1362 |
| Wan JCM, 2017, NAT REV CANCER, V17, P223, DOI 10.1038/nrc.2017.7 | 1160 |
| Garcia-Murillas I, 2015, SCI TRANSL MED, V7, DOI 10.1126/scitranslmed.aab0021 | 875 |
| Forshew T, 2012, SCI TRANSL MED, V4, DOI 10.1126/scitranslmed.3003726 | 869 |
| Alix-Pananieres C, 2016, CANCER DISCOV, V6, P479, DOI 10.1158/2159-8290.Cd-15-1483 | 821 |
| Heitzer E, 2015, CLIN CHEM, V61, P112, DOI 10.1373/clinchem.2014.222679 | 497 |
| Merker JD, 2018, J CLIN ONCOL, V142, P1242, DOI 10.5858/arpa.2018-0901-SA | 456 |

Supplementary Table S4 | The top 10 references with the most co-citations in this field

| Cited references (Author/year/journal/Volume/Page/DOI) | TC |
|---|---|
| Dawson SJ, 2013, NEW ENGL J MED, V368, P1199, DOI 10.1056/NEJMOA1213261 | 310 |
| Bettegowda C, 2014, SCI TRANSL MED, V6, DOI 10.1126/SCITRANSLMED.3007094 | 266 |
| Garcia-Murillas I, 2015, SCI TRANSL MED, V7, DOI 10.1126/SCITRANSLMED.AAB0021 | 228 |
| Diehl F, 2008, NAT MED, V14, P985, DOI 10.1038/NM.1789 | 189 |
| Murtaza M, 2013, NATURE, V497, P108, DOI 10.1038/NATURE12065 | 173 |

| | |
|---|---|
| Newman AM, 2014, NAT MED, V20, P552, DOI 10.1038/NM.3519 | 145 |
| Forshew T, 2012, SCI TRANSL MED, V4, DOI 10.1126/SCITRANSLMED.3003726 | 134 |
| Diaz LA, 2014, J CLIN ONCOL, V32, P579, DOI 10.1200/JCO.2012.45.2011 | 125 |
| Olsson E, 2015, EMBO MOL MED, V7, P1034, DOI 10.15252/EMMM.201404913 | 115 |
| Schwarzenbach H, 2011, NAT REV CANCER, V11, P426, DOI 10.1038/NRC3066 | 113 |
